# Efficacy and Safety Evaluation of Immune Checkpoint Inhibitors in Combination With Chemotherapy for Extensive Small Cell Lung Cancer: Real‐World Evidence

**DOI:** 10.1002/cam4.70480

**Published:** 2024-12-19

**Authors:** Yuta Yamanaka, Yukiko Okuno, Keisuke Kamisako, Yuta Okazaki, Kentaro Nakanishi, Yume Sanada, Kiyori Yoshida, Tatsuki Ikoma, Yuki Takeyasu, Utae Katsushima, Hiroshige Yoshioka, Takayasu Kurata

**Affiliations:** ^1^ Department of Thoracic Oncology Kansai Medical University Hospital Osaka Japan

**Keywords:** atezolizumab, durvalumab, extensive‐stage small cell lung cancer, first‐line treatment, immune checkpoint blockade, real‐world evidence

## Abstract

**Introduction:**

Extensive small cell lung cancer (ES‐SCLC) are currently managed using first‐line chemotherapy options, including atezolizumab (Atezo) plus etoposide and carboplatin (CE) or durvalumab (Durva) plus etoposide with either cisplatin (PE) or carboplatin (CE). However, a definitive distinction in therapeutic effects between Atezo and Durva in these regimens remains unestablished.

**Methods:**

We analyzed data from 100 patients diagnosed with ES‐SCLC who received immune checkpoint inhibitors (ICIs) as first‐line chemotherapy. Among them, 70 were administered Atezo + CE, 12 received Durva + PE, and 18 received Durva + CE. We assessed the efficacy of the two ICIs across various factors.

**Results:**

The progression‐free survival (PFS) and overall survival (OS) did not significantly differ between Atezo + CE and Durva + CE/PE as first‐line chemotherapy treatments for SCLC. We observed no significant differences in age, sex, performance status (PS), liver metastasis, bone metastasis, or platinum‐based agent usage between the treatment cohorts. However, a marked improvement in PFS and OS was observed in the solitary patient with brain metastasis treated with Atezo + CE.

**Conclusion:**

The primary distinction between these treatments was observed in the management of patients with brain metastasis. The literature lacks comparative studies on the effects of first‐line ICI treatment on the central nervous system, rendering our findings significant in clinical practice. Despite the retrospective nature of this study and the potential for various biases, we recommend the preferential use of Atezo + CE in patients with brain metastasis to potentially enhance prognosis.

## Graphical abstract

This is a comparison of 1st line Chemotherapy for small cell lung cancer. There was a difference in efficacy for brain metastasis.

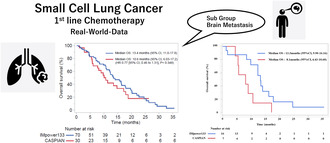

## 1 Introduction

Lung cancer remains a leading cause of mortality worldwide [1]. Particularly, small‐cell lung cancer (SCLC) is known for its aggressiveness, high recurrence rate, and increased morbidity and mortality. Most patients often present with this condition at an advanced stage, characterized by rapid growth and early metastasis, leading to a diagnosis of extensive‐stage SCLC (ES‐SCLC). Although SCLC accounts for only 10%–20% of all lung cancer cases, the 5‐year survival rate is low at 7% [2]. However, treatment approaches for small cell carcinoma have remained stagnant for over three decades. Historically, the first‐line therapy for ES‐SCLC consisted of a combination of platinum and etoposide [3], yielding objective response rates of 40%–70% [4]. Following the approval of nivolumab for the treatment of malignant melanoma in 2014 [5], immune checkpoint inhibitors (ICIs) have revolutionized cancer therapy, substantially improving treatment outcomes across various malignancies. Recently, we conducted two pivotal clinical trials assessing the efficacy of programmed cell death 1 (PD‐1)/programmed death‐ligand 1 (PD‐L1) inhibitors in combination with chemotherapy for ES‐SCLC in the first‐line setting. Atezolizumab (Atezo) gained approval in August 2019 for small‐cell carcinoma, and recent studies, such as the IMpower 133 trial, have reported improved progression‐free survival (PFS) and overall survival (OS) rates with the combined use of this ICI and chemotherapy [6]. Similarly, durvalumab (Durva) became available for ES‐SCLC in August 2020 and, similar to Atezo, it has shown comparable improvements in PFS and OS when combined with chemotherapy, as in the CASPIAN trial [7].

IMpower133, a double‐blind, Phase III, placebo‐controlled trial, investigated the efficacy of combining chemotherapy with Atezo as a first‐line treatment for ES‐SCLC. The primary endpoints of this trial were OS and PFS. Patients receiving Atezo demonstrated a median OS of 12.3 months compared to 10.3 months in the placebo group. Additionally, patients in the Atezo group experienced a median PFS of 5.2 months, whereas those in the placebo group had a median PFS of 4.3 months.

CASPIAN evaluated the combination of Durva with chemotherapy as a first‐line treatment for ES‐SCLC, with the primary endpoint being OS. The study showed a considerable improvement in median OS with the Durva combination, at 13.0 months compared to 10.3 months observed with chemotherapy alone. Additionally, patients receiving chemotherapy alone had a median PFS of 5.4 months compared with 5.1 months in those receiving chemotherapy plus Durva.

An additional analysis of IMpower133, known as the IMbrella A extension study, reported a 5‐year survival rate of 12% among patients treated with ICI plus chemotherapy. This improvement in the 5‐year survival rate in patients with SCLC with ICI reflects a similar trend observed in patients with non‐SCLC (NSCLC). However, it is worth noting that most clinical trials for ES‐SCLC have primarily focused on patients with favorable disease status, leading to stringent eligibility criteria and exclusion of many patients [8, 9, 10]. Therefore, further clinical information is warranted. Given the emerging role of ICIs in long‐term survival of patients with SCLC, we hypothesized that understanding their effectiveness in different patient populations will affect future prognostic outcomes. Consequently, in this study, we aimed to retrospectively analyze the treatment data of patients who received chemotherapy for ES‐SCLC at our hospital and compare real‐world outcomes between the IMpower133 and CASPIAN regimens.

## 2 Materials and Methods

### 2.1 Study Design

This retrospective study utilized the data of patients who received chemotherapy for ES‐SCLC at our hospital. Approval for the study was obtained from the institutional review board of our hospital, Kansai Medical University Ethics Review Center, on March 06, 2021, with the hospital reference number 2021306. The study was conducted in accordance with the principles of the Declaration of Helsinki. Owing to the retrospective analysis of anonymized patient data, the need for informed consent was waived.

### 2.2 Patients

This analysis was conducted exclusively at Kansai Medical University Hospital. We collected the data of 118 patients diagnosed with ES‐SCLC at our institution from August 2019, when Atezo was approved for insurance coverage, to August 2023. The inclusion criterion was patients who received a combination of chemotherapy and ICI as the first‐line chemotherapy. The exclusion criterion was patients whose first‐line treatment did not include an ICI.

### 2.3 Endpoints

We extracted the following information from the electronic medical records: age, sex, Eastern Cooperative Oncology Group Performance Status (ECOG‐PS), smoking history, metastasis status, PFS, OS, efficacy parameters such as objective response rate (ORR) and disease control rate (DCR), toxicity data including adverse events (AEs) and immune‐related adverse events (irAEs), results of blood investigations, details of regimen administered, and dosage at initiation. The primary endpoint was OS, and the secondary endpoints were PFS, ORR, and safety evaluations. Subgroup analyses were conducted for OS and PFS based on the data collected.

### 2.4 Statistical Analysis

PFS was defined as the duration from treatment commencement to either disease progression or death and OS was defined as the period from treatment initiation to death. PFS and OS were analyzed using the Kaplan–Meier method, with median values and corresponding 95% CIs calculated via Brookmeyer–Crowley method. Categorical variables are presented as number and percentage, and comparisons were made using the chi squared test or Fisher exact test. Between‐group analyses were conducted using the log‐rank test and Cox proportional hazards regression model, with results expressed as HR accompanied by 95% CI. Statistical significance was set at *p* < 0.05. All analyses were performed using JMP Pro V.17 (SAS Institute Inc., Cary, NC, USA).

Tumor response was categorized as complete response (CR), partial response (PR), stable disease (SD), progressive disease (PD), or not evaluable (NE), following the Response Evaluation Criteria in Solid Tumors version 1.1 [11]. AEs and irAEs were evaluated using the Common Terminology Criteria for Adverse Events (version 5.0) [12]. The evaluation period for AEs and irAEs corresponded to the duration of PFS from treatment initiation to either tumor progression or death.

## 3 Results

**FIGURE 1:**
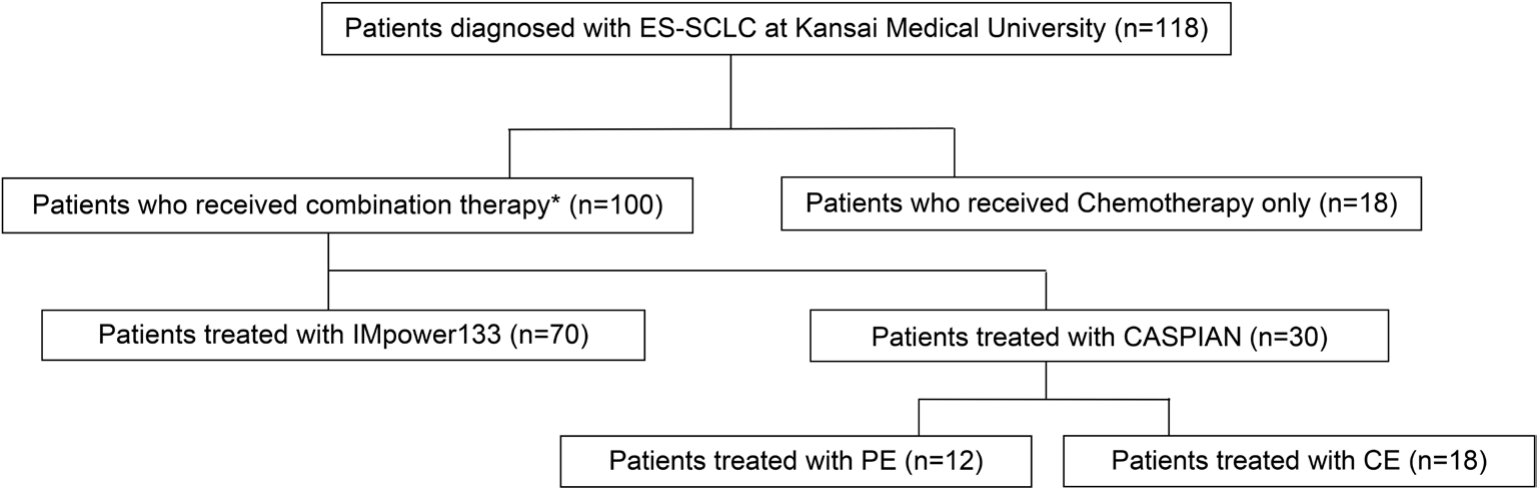
Patients enrollment flowchart. *Combination of PD‐L1 blockade and chemotherapy in the first line treatment of ES‐SCLC. ES‐SCLC, extensive‐stage small‐cell lung cancer; OS, Overall Survival; PFS, Progression‐Free Survival.

Figure 1 depicts the patient selection process. Among the 118 patients, 100 received combined treatment with PD‐L1 blockade and chemotherapy as the first‐line therapy for ES‐SCLC. The remaining 18 patients did not receive ICIs due to interstitial pneumonia or personal preference. Notably, the decision to administer combined PD‐L1 blockade and chemotherapy as the first‐line treatment for ES‐SCLC was made based on the attending physician's discretion. A total of 70 patients were treated with the Impower133 regimen and 30 with the CASPIAN regimen. In the CASPIAN regimen group, 12 patients received cisplatin and 18 received carboplatin. Following the initial treatment, maintenance therapy with PD‐L1 blockade was administered until disease progression, death, or unacceptable toxicity was observed among the treated patients.

The patient characteristics for each group are presented in Table 1. The median age was 74.5 years for the IMpower133 group and 76.0 years for the CASPIAN group, showing no significant difference. All patients receiving cisplatin in the CASPIAN regimen group were under 75 years old and had preserved cardiac and renal functions, as our hospital policy restricts the use of cisplatin to patients under 75 years of age with these criteria. There was no significant difference in the sex ratio between the groups. IMpower133 had a higher percentage of patients with an ECOG‐PS of 2. The proportion of patients with brain and liver metastases was similar between the groups, whereas patients with bone metastases were more likely to receive the CASPIAN regimen. First, we analyzed the data on the efficacy and safety of the IMpower133 and CASPIAN regimens. The median follow‐up for the population was 10.9 months (range: 0.7–37.8 months).

**TABLE 1:** Patient characteristics.

*n* (%)	IMpower133	CASPIAN			*p*
	CBDCA + ETP + Atezo (*n* = 70)	CASPIAN Total (*n* = 30)	CBDCA + ETP + Dur (*n* = 18)	CDDP + ETP + Dur (*n* = 12)	
Age, median(range) years	74.5 (34–85)	76.0 (46–88)	78.5 (73–88)	64.5 (46–73)	0.954
≤ 75	41 (59)	14 (47)	2 (11)	12 (100)	
< 75	29 (41)	16 (53)	16 (89)	0 (0)	
Sex					
Male	49 (70)	23 (76)	16 (89)	7 (58)	0.462
Female	21 (30)	7 (24)	2 (11)	5 (42)	
ECOG PS					
0	38 (54)	15 (50)	9 (50)	6 (50)	0.339
1	22 (31)	13 (43)	8 (45)	5 (42)	
2	10 (15)	2 (7)	1 (5)	1 (8)	
Metastasis					
Brain+	16 (23)	7 (24)	1 (5)	6 (50)	0.958
Brain−	54 (77)	23 (76)	17 (95)	6 (50)	
Liver+	17 (24)	8 (27)	2 (11)	6 (50)	0.449
Liver−	53 (76)	22 (73)	16 (89)	6 (50)	
Bone+	19 (27)	14 (47)	6 (33)	8 (67)	0.057
Bone−	51 (73)	16 (53)	12 (67)	4 (33)	

*Notes:* IMpower133 had a higher rate of patients with an ECOG‐PS of 2, and CASPIAN had a higher rate of patients with bone metastases.

Abbreviations: Atezo, Atezolizumab; CBDCA, Carboplatin; CDDP, Cisplatin; Dur, Durvalumab; ECOG PS, Eastern Cooperative Oncology Group Performance Status; ETP, Etoposide.

**FIGURE 2:**
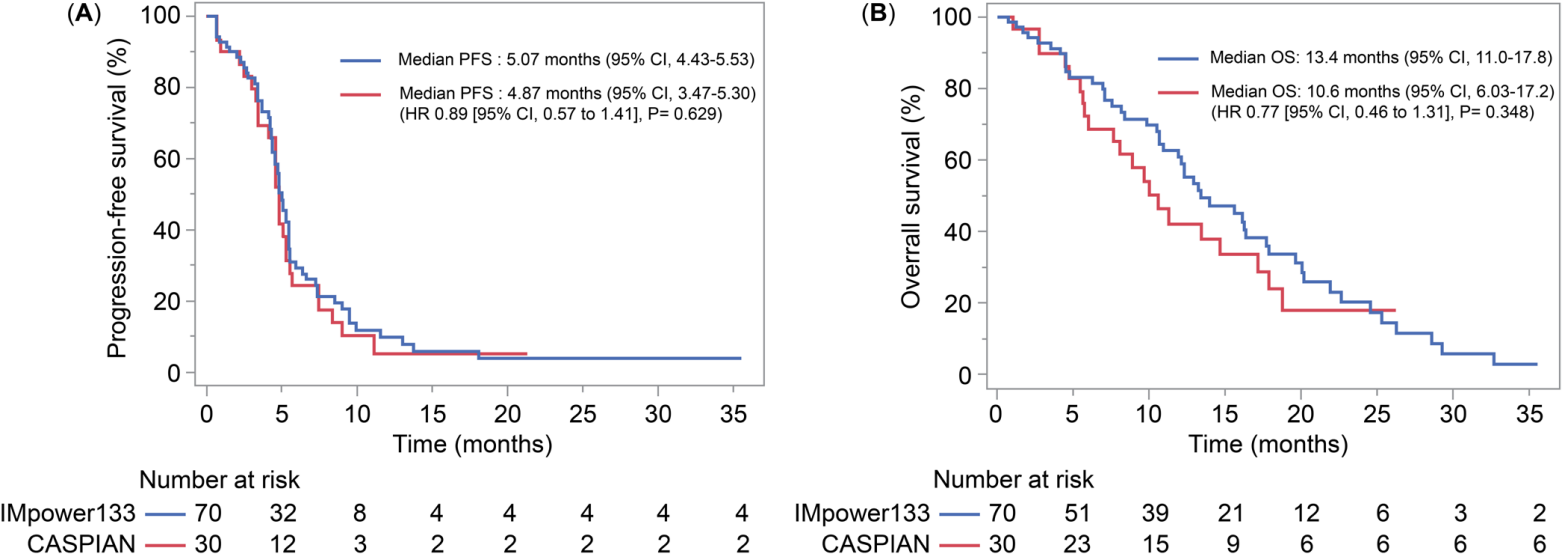
Kaplan–Meier estimates of (A) PFS and (B) OS in patients who received the IMpower133 and CASPIAN regimens. OS, overall survival; PFS, progression‐free survival.

The median PFS and median OS with IMpower133 regimen were 5.07 (95% CI, 4.43–5.53) and 13.43 (95% CI, 10.97–17.77) months, whereas those with CASPIAN regimen were 4.87 (95% CI, 3.47–5.30) and 10.6 (95% CI, 6.03–17.18) months, respectively. These findings were consistent with the clinical trial data of IMpower133 and CASPIAN. Figure 2 illustrates a comparison of the datasets from our hospital, indicating no significant difference in PFS and OS between IMpower133 and CASPIAN for first‐line chemotherapy against SCLC (PFS: HR, 0.89; 95% CI, 0.57–1.41; *p* = 0.629; OS: HR, 0.77; 95% CI, 0.46–1.31; *p* = 0.348).

**FIGURE 3:**
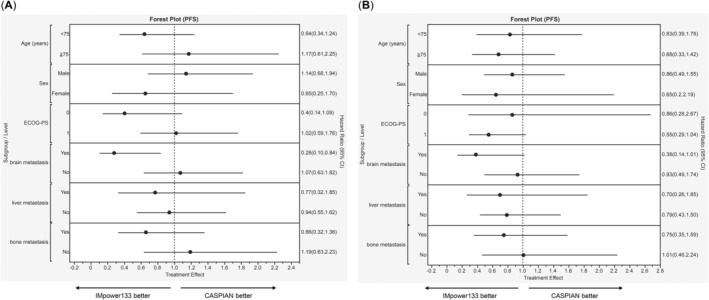
Forest plot of (A) PFS and (B) OS subgroup analyses. OS, overall survival; PFS, progression‐free survival.

**FIGURE 4:**
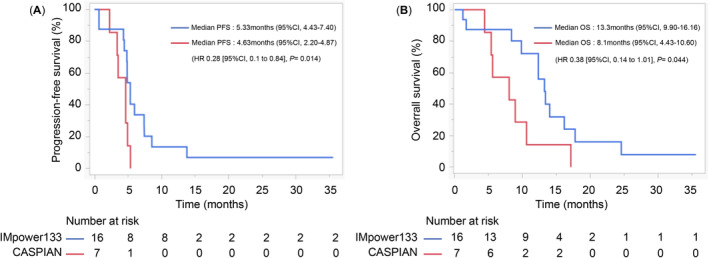
Kaplan–Meier estimates of PFS and OS in patients with brain metastases. OS, overall survival; PFS, progression‐free survival.

The subgroup analysis results are presented in Figure 3, categorized by age, sex, ECOG‐PS score, and presence of metastasis. Notably, significant differences were observed only for brain metastasis. Patient characteristics for brain metastases are detailed in Table 2. The CASPIAN group exhibited a younger age and better ECOG‐PS scores than the IMpower133 group. Figure 4 illustrates the PFS and OS rates. The PFS and OS of IMpower133/CASPIAN were 5.33/4.63 (HR, 0.29; 95% CI, 0.10–0.84; *p* = 0.014) and 13.3/8.0 months (HR, 0.38; 95% CI, 0.14–1.01; *p* = 0.044), respectively. These findings suggest that IMpower133 regimen outperformed CASPIAN regimen in patients with brain metastases, demonstrating significant differences in both PFS and OS.

**TABLE 2:** Characteristics of patients with brain metastases.

*n* (%)	IMpower133	CASPIAN		
	CBDCA + ETP + Atezo (*n* = 16)	CASPIAN Total (*n* = 7)	CBDCA + ETP + Dur (*n* = 1)	CDDP + ETP + Dur (*n* = 6)
Age, median(range) years	72.5 (58–80)	69.0 (46–80)	80 (−)	66.0 (46–70)
≤ 75	10 (63)	6 (86)	0 (0)	6 (100)
< 75	6 (47)	1 (14)	1 (100)	0 (0)
Sex				
Male	13 (81)	5 (71)	1 (100)	4 (67)
Female	3 (19)	2 (29)	0 (0)	2 (33)
ECOG PS				
0	7 (44)	3 (43)	0 (0)	3 (50)
1	7 (44)	4 (57)	1 (100)	3 (50)
2	2 (12)	0 (0)	0 (0)	0 (0)

*Notes:* The CASPIAN group exhibited a younger age and better ECOG‐PS scores than the IMpower133 group.

Abbreviations: Atezo, Atezolizumab; CBDCA, Carboplatin; CDDP, Cisplatin; Dur, Durvalumab; ECOG PS, Eastern Cooperative Oncology Group Performance Status; ETP, Etoposide.

**FIGURE 5:**
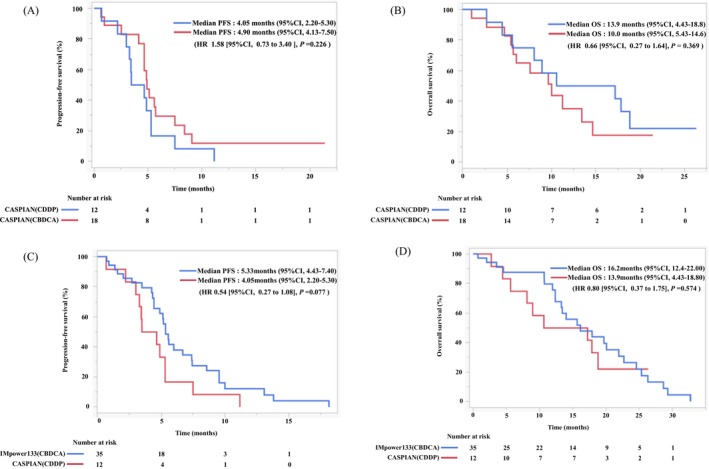
Kaplan–Meier estimates of (A) PFS and (B) OS in patients treated with CDDP or CBDCA in CASPIAN. Kaplan–Meier estimates of (C) PFS and (D) OS in patients treated with IMpower133 or CDDP in CASPIAN under 75 years of age. CBDCA, carboplatin; CDDP, cisplatin; OS, overall survival; PFS, progression‐free survival.

To compare the efficacy of platinum agents, the initial step was to assess the differences between cisplatin (CDDP) and carboplatin (CBDCA) in the CASPIAN regimen. Patient characteristics are outlined in Table 1; PFS and OS are depicted in Figure 5A,B, respectively. The PFS and OS in patients receiving CDDP/CBDCA in CASPIAN were 4.05/4.9 (HR: 1.58; 95% CI, 0.73–3.40; *p* = 0.226) and 13.9/10.0 months (HR: 0.66; 95% CI, 0.27–1.64; *p* = 0.369), respectively. No significant differences were observed in PFS and OS in both groups. Age was identified as the primary factor influencing the reversal of PFS and OS in both groups. Consequently, the comparison between the platinum agents in IMpower133 and CASPIAN was limited to patients aged < 75 years treated with CDDP in CASPIAN. Figure 5C,D shows the PFS and OS in patients under 75 years of age in both groups. The PFS and OS in IMpower133/CASPIAN were 5.33/4.05 (HR: 0.54; 95% CI, 0.27–1.08; *p* = 0.077) and 16.2/13.9 months (HR: 0.80; 95% CI, 0.37–1.75; *p* = 0.337), respectively. There were no significant differences in PFS and OS between the groups.

The treatment response is shown in Table 3. The combination of chemotherapy and Atezo in the IMpower 133 trial resulted in the following: 0 (0%) CR, 53 (75.7%) PR, 10 (14.2%) SD, 6 (8.57%) PD, and 1 (1.42%) NE, yielding an ORR and DCR of 75.7% and 90.0%, respectively. In CASPIAN, the distribution was as follows: 0 (0%) CR, 23 (76.6%) PR, 5 (16.6%) SD, 2 (6.66%) PD, and 0 (0%) NE, with an ORR and DCR of 76.6% and 93.3%, respectively. There were no significant differences in treatment response, PFS, and OS between the regimens.

**TABLE 3:** Treatment response.

*n* (%)	IMpower133	CASPIAN
	CBDCA + ETP + Atezo (*n* = 70)	CASPAIN Total (*n* = 30)
CR	0 (0)	0 (0)
PR	53 (76)	23 (77)
SD	10 (14)	5 (16)
PD	6 (8)	2 (7)
NE	1 (1)	0 (0)
ORR	53 (76)	23 (77)
DCR	63 (90)	28 (93)

Abbreviations: Atezo, Atezolizumab; CBDCA, Carboplatin; CR, Complete response; DCR, Disease control rate; ETP, Etoposide; ORR, Overall response rate; PD, Progressive disease, NE, Not evaluable; PR, Partial response; SD, Stable disease.

The AEs and irAEs are summarized in Table 4. A higher incidence of AEs was observed in CASPIAN. The increased use of CASPIAN in our hospital, likely due to participation in clinical trials, could explain the higher number of medical record entries. Most treatment‐related hematological toxicities of Grades 3–5 in both IMpower133 and CASPIAN were attributed to myelosuppression associated with the administration of cytotoxic anticancer drugs, including leukopenia and neutropenia. Febrile neutropenia (FN) occurred in 16 cases (22.8%) in IMpower133 and 10 cases (33.3%) in CASPIAN. These data closely align with the actual clinical trial data for IMpower133 and CASPIAN. Grade 2 or higher irAEs in IMpower133 included five cases of skin rash and two cases of thyroid dysfunction of Grade 2, three cases of adrenal insufficiency of Grade 3, and two cases of interstitial lung disease of Grade 4. Grade 2 or higher irAEs in CASPIAN included two Grade 2 cases of thyroid dysfunction, one Grade 2 case of interstitial lung disease, one case of adrenal insufficiency of Grade 3, and two cases of interstitial lung disease of Grade 5.

**TABLE 4:** Adverse events.

Data on treatment‐related AEs or irAEs			
		IMpower133	CASPIAN
Number of patients	*n* (%)	70	30
TR‐hematologic toxicity	Grades 3–5	59 (84)	25 (83)
	Grade 3	26 (37)	6 (20)
	Grade 4	33 (47)	19 (63)
	Grade 5	0 (0)	0 (0)
TR‐non‐hematologic toxicity	Grades 3–5	18 (26)	20 (67)
	Grade 3	17 (24)	16 (53)
	Grade 4	0 (0)	2 (7)
	Grade 5	1 (1)	2 (7)
TR‐irAE	Grades 2–5	12 (17)	6 (20)
	Grade 2	7 (10)	2 (7)
	Grade 3	3 (4)	2 (7)
	Grade 4	2 (3)	0 (0)
	Grade 5	0 (0)	2 (7)

Abbreviations: AEs, Adverse events; irAEs: Immune‐related adverse events; TR, treatment‐related.

Regarding Grade 5 AEs or irAEs, there was one case (1.4%) in IMpower133 and four cases (14%) in CASPIAN. Pneumonia accounted for all Grade 5 AEs and interstitial lung diseases accounted for all irAEs.

## 4 Discussion

Our findings suggest that selecting first‐line treatment for ES‐SCLC based on age, sex, liver metastasis, bone metastasis, or platinum agents may not be necessary. However, the IMpower133 regimen appears more effective in patients with brain metastasis. To our knowledge, there are no data on SCLC indicating emerging differences between treatment regimens that would warrant individualization based on patient characteristics.

In clinical practice, there is often no clear distinction between the IMpower133 and CASPIAN regimens, and currently, the choice of platinum agent or presence of brain metastasis often dictates the regimen used. The CASPIAN regimen is often preferred owing to its flexibility in offering a choice between CDDP or CBDCA platinum agents and its suitability for patients with symptomatic or untreated brain metastases; thus, the CASPIAN trial provides valuable treatment data for this subgroup. In this milieu, we aimed to discuss these differences, incorporating insights from our hospital's study data.

Immunotherapy for SCLC has been explored in various trials since the NCT01450761 trial introduced ipilimumab in 2015 [13]. Notably, several trials have investigated immunotherapy as first‐line, maintenance, or subsequent‐line treatment, with a few reporting positive outcomes. Besides ipilimumab in 2015, Atezo in 2018, Durva in 2019, pembrolizumab in 2020 [14], and adebrelimab [15] and serplulimab [16] in 2022 have been utilized as first‐line treatments for SCLC. Among these, Atezo (IMpower133) and Durva (CASPIAN) are currently covered by insurance and available for first‐line use. Currently, there are no trials directly comparing the IMpower133 and CASPIAN regimens as first‐line treatments in combination with chemotherapy and ICIs for ES‐SCLC. Similar to the approach in NSCLC treatment based on PD‐L1 status, the unique characteristics of Atezo and Durva are expected to personalize treatments for SCLC. As there is no clear distinction in the therapeutic effects of Atezo or Durva in first‐line chemotherapy, and it is not practical to apply ES‐SCLC treatment uniformly to individual patients, the choice of treatment in the real world is often challenging.

Regarding the difference in platinum agents, a meta‐analysis comparing CDDP and CBDCA did not find any difference in OS and PFS before ICI treatment, and there was no clear superiority between the drugs [17]. Our study results corroborated these findings, indicating no significant difference between the drugs even after ICI administration. Furthermore, the reasons for the effectiveness of the IMpower133 regimen remain unclear. However, we speculate that the treatment schedule plays a role in the overall efficacy of the IMpower133 regimen, not just in the context of brain metastases.

Here, the FN rates tended to be higher in the CASPIAN group. This trend was also noted in the Japanese subsets of the IMpower133 and CASPIAN studies, with FN rates of 11.9% [18] and 33.3% [19], respectively. Research has shown that FN can lead to treatment delays and affect dose intensity, which is crucial in ES‐SCLC management. For instance, in the CASPIAN regimen, the dosing interval of Durva changes from every 3 to 4 weeks during maintenance therapy. Thus, given the known effect of dosing intervals on outcomes, as observed in other studies, such as OS in CheckMate 451 [20] and PFS in Pacific [21], we cannot rule out the possibility that the change in the dosing interval influenced the treatment efficacy.

The reasons for not adding ICI in the initial chemotherapy of SCLC at various centers were predominantly age, ECOG‐PS, and ILD. Although ILD was not specifically evaluated in this study, it was considered with respect to age and ECOG‐OS. SCLC typically progresses rapidly, leading to a decline in ECOG‐PS. Research has shown that the older the patient, the more pronounced the decline. However, chemotherapy can often improve ECOG‐PS by targeting the underlying tumors. A previous study has demonstrated the safety and efficacy of chemotherapy, including ICIs, across different age groups and ECOG‐PS statuses [22]. Consequently, we recommend a combination of chemotherapy with ICIs for elderly patients and those with poor ECOG‐PS based on efficacy.

While several ES‐SCLC cases with long‐term survival have been recorded at our hospital, identifying patients who can achieve such outcomes remains unclear. The use of ICIs in ES‐SCLC treatment has been explored in various trials since March 2015, when the NCT01450761 trial reported the addition of ipilimumab to chemotherapy. Despite these efforts, no biomarker, such as PD‐L1, for NSCLC has been identified to reliably predict the efficacy of ICIs in ES‐SCLC. Although the tumor mutation burden (TMB) is generally elevated in ES‐SCLC [23], it may not reliably predict treatment response, as demonstrated in the KEYNOTE 028/189 trials [24, 25]. Additionally, ES‐SCLC typically exhibits a low proportion of tumor‐infiltrating lymphocytes [26], which may explain the challenge in achieving a therapeutic effect with ICIs, even in cases with high TMB. Consequently, enhancing the antitumor effect in ES‐SCLC may require stimulating the activation of the immune cycle [27], as evidenced by the results of CheckMate331 [28]. The combination of anti‐cytotoxic T‐lymphocyte‐associated protein 4 (CTLA4) blockade in the first line or maintenance therapy settings has been investigated in studies such as NCT01450761 and CheckMate 451, both of which yielded negative studies. However, the landscape of first‐line treatment in SCLC may evolve based on the outcomes of anti‐vascular endothelial growth factor blockade studies. In the absence of definitive treatment, real‐world data, including findings from studies such as ours, will be important in determining the optimal treatment approach for individual patients with ES‐SCLC. We hope that the insights obtained in this study will contribute to clinical decision‐making and ultimately benefit patients in practice.

This study has some limitations. First, the retrospective nature of the study introduced various biases. The reporting of adverse events, for example, relied on the descriptions provided by the attending physicians, which may vary in detail and accuracy. Second, there was a discrepancy in the number of cases between the IMpower133 and CASPIAN groups, which could potentially influence the results. Increasing the sample size by collecting data from multiple facilities rather than focusing solely on our hospital may mitigate this limitation. To address potential biases and enhance the robustness of the analysis, the use of propensity score matching could be considered to balance baseline characteristics between groups and enhance the validity of the findings. This would help to distinguish true statistically significant differences from those arising from real‐world data variability.

## 5 Conclusion

No significant differences in OS and PFS were observed between the IMpower133 and CASPIAN groups. However, in clinical practice, the choice between these regimens often depends on the use of platinum‐based drugs and presence or absence of brain metastasis. In the subgroup analyses conducted, the IMpower133 regimen demonstrated effectiveness in patients with lung cancer and brain metastasis. While Atezo + CE lacks specific data regarding brain metastases, Durva is commonly used in clinical practice. Nonetheless, Atezo + CE may be more efficacious in treating brain metastases. Although various factors must be considered when selecting between the regimens, our findings suggest that the preferential use of Atezo + CE may improve life expectancy of patients with SCLC having brain metastasis and an unfavorable prognosis.

## Author Contributions

**Yuta Yamanaka:** conceptualization (lead), data curation (lead), formal analysis (lead), investigation (lead), methodology (lead), resources (lead), validation (lead), visualization (lead), writing – original draft (lead), writing – review and editing (lead). **Yukiko Okuno:** investigation (supporting), resources (supporting). **Keisuke Kamisako:** investigation (supporting), resources (supporting). **Yuta Okazaki:** investigation (supporting), resources (supporting). **Kentaro Nakanishi:** investigation (supporting), resources (supporting). **Yume Sanada:** investigation (supporting), resources (supporting). **Kiyori Yoshida:** investigation (supporting), resources (supporting). **Tatsuki Ikoma:** investigation (supporting), resources (supporting). **Yuki Takeyasu:** investigation (supporting), resources (supporting). **Utae Katsushima:** investigation (supporting), resources (supporting). **Hiroshige Yoshioka:** investigation (supporting), resources (supporting). **Takayasu Kurata:** investigation (equal), resources (equal), supervision (equal), writing – original draft (supporting), writing – review and editing (supporting).

## Ethics Statement

Approval for the study was obtained from the Institutional Review Board of our hospital on March 06, 2021, with the hospital reference number 2021306.

## Consent

Owing to the retrospective analysis of anonymized patient data, the need for informed consent was waived.

## Conflicts of Interest

Takayasu Kurata received grants from MSD, Astra Zeneca, Amgen, Boehringer Ingelheim, Daiichi Sankyo Pharmaceutical, Takeda Pharmaceutical, and Bristol Myers Squibb and honoraria for lecture from Astra Zeneca, Ono Pharmaceutical, MSD, Nippon Kayaku, Takeda Pharmaceutical, Eli Lilly, Bristol Myers Squibb, Chugai Pharmaceutical, and Pfizer. Hiroshige Yoshioka received honoraria for lecture fees from Boehringer Ingelheim, Chugai Pharmaceutical, Nippon Kayaku, Taiho Pharmaceutical, Eli Lilly, Takeda Pharmaceutical, and Bristol Myers Squibb. The other authors declare no conflicts of interest.

## Data Availability

The data that support the findings of this study are available from the corresponding author upon reasonable request.

## References

[1] H. Sung , J. Ferlay , R. L. Siegel , et al., “Global Cancer Statistics 2020: GLOBOCAN Estimates of Incidence and Mortality Worldwide for 36 Cancers in 185 Countries,” CA: A Cancer Journal for Clinicians 71, no. 3 (2021): 209–249. 33538338 10.3322/caac.21660

[2] R. M. Huber and A. Tufman , “Update on small cell lung cancer management,” Breathe 8 (2012): 314–330.

[3] W. K. Evans , F. A. Shepherd , R. Feld , D. Osoba , P. Dang , and G. Deboer , “VP‐16 and Cisplatin as First‐Line Therapy for Small‐Cell Lung Cancer,” Journal of Clinical Oncology 3, no. 11 (1985): 1471–1477. 2997406 10.1200/JCO.1985.3.11.1471

[4] D. M. Jackman and B. E. Johnson , “Small‐cell lung cancer,” Lancet 366, no. 9494 (2005): 1385–1396. 16226617 10.1016/S0140-6736(05)67569-1

[5] C. Robert , G. V. Long , B. Brady , et al., “Nivolumab in Previously Untreated Melanoma Without BRAF Mutation,” New England Journal of Medicine 372, no. 4 (2015): 320–330. 25399552 10.1056/NEJMoa1412082

[6] L. Horn , A. S. Mansfield , A. Szczęsna , et al., “First‐Line Atezolizumab Plus Chemotherapy in Extensive‐Stage Small‐Cell Lung Cancer,” New England Journal of Medicine 379, no. 23 (2018): 2220–2229. 30280641 10.1056/NEJMoa1809064

[7] L. Paz‐Ares , “Durvalumab Plus Platinum‐Etoposide Versus Platinum‐Etoposide in First‐Line Treatment of Extensive‐Stage Small‐Cell Lungcancer (CASPIAN): A Randomised, Controlled, Open‐Label, Phase 3 Trial,” Lancet 394 (2019): 1929–1939. 31590988 10.1016/S0140-6736(19)32222-6

[8] M. Q. Baggstrom , S. N. Waqar , A. K. Sezhiyan , et al., “Barriers to Enrollment in Non‐small Cell Lung Cancer Therapeutic Clinical Trials,” Journal of Thoracic Oncology 6, no. 1 (2011): 98–102. 21150469 10.1097/JTO.0b013e3181fb50d8

[9] H. Kawachi , D. Fujimoto , T. Morimoto , et al., “Clinical Characteristics and Prognosis of Patients With Advanced Non‐small‐Cell Lung Cancer Who Are Ineligible for Clinical Trials,” Clinical Lung Cancer 19, no. 5 (2018): e721–e734. 29934133 10.1016/j.cllc.2018.05.014

[10] L. Horn , V. L. Keedy , N. Campbell , et al., “Identifying Barriers Associated With Enrollment of Patients With Lung Cancer Into Clinical Trials,” Clinical Lung Cancer 14, no. 1 (2013): 14–18. 22591607 10.1016/j.cllc.2012.03.008

[11] L. H. Schwartz , S. Litière , E. de Vries , et al., “RECIST 1.1‐Update and Clarification: From the RECIST Committee,” European Journal of Cancer 62 (2016): 132–137. 27189322 10.1016/j.ejca.2016.03.081 PMC5737828

[12] National Cancer Institute , “Common Terminology Criteria for Adverse Events(CTCAE), version 5.0,” 2017.

[13] M. Reck , A. Luft , A. Szczesna , et al., “Phase III Randomized Trial of Ipilimumab Plus Etoposide and Platinum Versus Placebo Plus Etoposide and Platinum in Extensive‐Stage Small‐Cell Lung Cancer,” Journal of Clinical Oncology 34, no. 31 (2016): 3740–3748. 27458307 10.1200/JCO.2016.67.6601

[14] C. M. Rudin , M. M. Awad , A. Navarro , et al., “Pembrolizumab or Placebo Plus Etoposide and Platinum as First‐Line Therapy for Extensive‐Stage Small‐Cell Lung Cancer: Randomized, Double‐Blind, Phase III KEYNOTE‐604 Study,” Journal of Clinical Oncology 38, no. 21 (2020): 2369–2379. 32468956 10.1200/JCO.20.00793 PMC7474472

[15] J. Wang , C. Zhou , W. Yao , et al., “Adebrelimab or Placebo Plus Carboplatin and Etoposide as First‐Line Treatment for Extensive‐Stage Small‐Cell Lung Cancer (CAPSTONE‐1): A Multicentre, Randomised, Double‐Blind, Placebo‐Controlled, Phase 3 Trial,” Lancet Oncology 23, no. 6 (2022): 739–747. 35576956 10.1016/S1470-2045(22)00224-8

[16] Y. Cheng , L. Han , L. Wu , et al., “Effect of First‐Line Serplulimab vs Placebo Added to Chemotherapy on Survival in Patients With Extensive‐Stage Small Cell Lung Cancer: The ASTRUM‐005 Randomized Clinical Trial,” Journal of the American Medical Association 328, no. 12 (2022): 1223–1232. 36166026 10.1001/jama.2022.16464 PMC9516323

[17] A. Rossi , M. di Maio , P. Chiodini , et al., “Carboplatin‐ Or Cisplatin‐Based Chemotherapy in First‐Line Treatment of Small‐Cell Lung Cancer: The COCIS Meta‐Analysis of Individual Patient Data,” Journal of Clinical Oncology 30, no. 14 (2012): 1692–1698. 22473169 10.1200/JCO.2011.40.4905

[18] M. Nishio , S. Sugawara , S. Atagi , et al., “Subgroup Analysis of Japanese Patients in a Phase III Study of Atezolizumab in Extensive‐Stage Small‐Cell Lung Cancer (IMpower133),” Clinical Lung Cancer 20 (2019): 469–476. 31466854 10.1016/j.cllc.2019.07.005

[19] K. Hotta , M. Nishio , H. Saito , et al., “First‐Line Durvalumab Plus Platinum‐Etoposide in Extensive‐Stage Small‐Cell Lung Cancer: CASPIAN Japan Subgroup Analysis,” International Journal of Clinical Oncology 26, no. 6 (2021): 1073–1082. 33826027 10.1007/s10147-021-01899-8 PMC8134304

[20] T. K. Owonikoko , K. Park , R. Govindan , et al., “Nivolumab and Ipilimumab as Maintenance Therapy in Extensive‐Disease Small‐Cell Lung Cancer: CheckMate 451,” Journal of Clinical Oncology 39, no. 12 (2021): 1349–1359. 33683919 10.1200/JCO.20.02212 PMC8078251

[21] D. R. Spigel , C. Faivre‐Finn , J. E. Gray , et al., “Five‐Year Survival Outcomes From the PACIFIC Trial: Durvalumab After Chemoradiotherapy in Stage III Non‐small‐Cell Lung Cancer,” Journal of Clinical Oncology 40, no. 12 (2022): 1301–1311. 35108059 10.1200/JCO.21.01308 PMC9015199

[22] T. Takeda , T. Yamada , Y. Kunimatsu , et al., “Age‐Stratified Analysis of First‐Line Chemoimmunotherapy for Extensive‐Stage Small Cell Lung Cancer: Real‐World Evidence From a Multicenter Retrospective Study,” Cancers (Basel) 15, no. 5 (2023): 1543. 36900334 10.3390/cancers15051543 PMC10001399

[23] L. B. Alexandrov , S. Nik‐Zainal , D. C. Wedge , et al., “Signatures of Mutational Processes in Human Cancer,” Nature 500, no. 7463 (2013): 415–421. 23945592 10.1038/nature12477 PMC3776390

[24] P. A. Ott , E. Elez , S. Hiret , et al., “Pembrolizumab in Patients With Extensive‐Stage Small‐Cell Lung Cancer: Results From the Phase Ib KEYNOTE‐028 Study,” Journal of Clinical Oncology 35, no. 34 (2017): 3823–3829. 28813164 10.1200/JCO.2017.72.5069

[25] A. Marabelle , D. T. Le , P. A. Ascierto , et al., “Efficacy of Pembrolizumab in Patients With Noncolorectal High Microsatellite Instability/Mismatch Repair‐Deficient Cancer: Results From the Phase II KEYNOTE‐158 Study,” Journal of Clinical Oncology 38, no. 1 (2020): 1–10. 31682550 10.1200/JCO.19.02105 PMC8184060

[26] Y. Şenbabaoğlu , R. S. Gejman , A. G. Winer , et al., “Tumor Immune Microenvironment Characterization in Clear Cell Renal Cell Carcinoma Identifies Prognostic and Immunotherapeutically Relevant Messenger RNA Signatures,” Genome Biology 17, no. 1 (2016): 231. 27855702 10.1186/s13059-016-1092-z PMC5114739

[27] D. S. Chen and I. Mellman , “Oncology Meets Immunology: The Cancer‐Immunity Cycle,” Immunity 39, no. 1 (2013): 1–10. 23890059 10.1016/j.immuni.2013.07.012

[28] D. R. Spigel , D. Vicente , T. E. Ciuleanu , et al., “Second‐Line Nivolumab in Relapsed Small‐Cell Lung Cancer: CheckMate 331,” Annals of Oncology 32, no. 5 (2021): 631–641. 33539946 10.1016/j.annonc.2021.01.071

